# Factors Influencing Running Velocity at Lactate Threshold in Male and Female Runners at Different Levels of Performance

**DOI:** 10.3389/fphys.2020.585267

**Published:** 2020-11-04

**Authors:** Eva Maria Støa, Jan Helgerud, Bent R. Rønnestad, Joar Hansen, Stian Ellefsen, Øyvind Støren

**Affiliations:** ^1^Department of Sports, Physical Education and Outdoor Studies, University of South-Eastern Norway, Bø, Norway; ^2^Department of Circulation and Medical Imaging, Norwegian University of Science and Technology, Trondheim, Norway; ^3^Myworkout, Medical Rehabilitation Clinic, Trondheim, Norway; ^4^Section for Health and Exercise Physiology, Institute of Public Health and Sport Sciences, Inland Norway University of Applied Sciences, Lillehammer, Norway

**Keywords:** lactate threshold, running performance, maximal aerobic speed, long-distance running, lactate threshold training, percentage of maximal oxygen uptake, velocity at LT

## Abstract

**Background:**

The primary aim was to examine the relationship between lactate threshold (LT) expressed as percentage of maximal oxygen uptake (VO_2max_) and running velocity at LT (LT_V_). A secondary aim was to investigate to what extent VO_2max_, oxygen cost of running (C_R_), and maximal aerobic speed (MAS) determined LT_V_. A third aim was to investigate potential differences in LT and LT_V_ between elite, national and recreational runners, as well as possible gender differences regarding VO_2max_, C_R_, LT, and LT_V_.

**Methods:**

Seventy-five competitive runners (37 males and 38 females) with an average VO_2max_ of 63.0 ± 9.3 mL⋅kg^–1^⋅min^–1^, and an average LT_V_ of 13.6 ± 2.3 km⋅h^–1^ were tested for VO_2max_, LT, LT_V_, MAS, and C_R_.

**Results:**

Lactate threshold did not correlate with LT_V_. With an *r* – value of 0.95 (*p* < 0.001) and a standard error of estimate of 4.0%, the product of MAS and individual LT determined 90% of LT_V_, outside a range of ±0.27 km⋅h^–1^. LT_V_ increased with higher performance level. However, LT did not differ between elite, national and recreational runners. Female runners had 2.5% higher LT, 8% lower LT_V_, and 21% lower VO_2max_, but 9% better C_R_ than male runners.

**Conclusion:**

Lactate threshold did not correlate with LT_V_. The product of MAS and LT correlated strongly with LT_V_. There were no differences between elite, national and recreational runners regarding LT, but female runners had higher LT than the male runners. Female runners at the same relative performance level had lower LT_V_ and VO_2max_, but better C_R_ than male runners.

## Introduction

Long-distance running performance is mainly determined by maximal oxygen uptake (VO_2max_), fractional utilization of VO_2max_ or lactate threshold (LT), and oxygen cost of running (C_R_) (Conley and Krahenbuhl, 1980; Pate and Kriska, 1984; Rabadán et al., 2011). LT has been defined as the highest work intensity where there is a balance between lactate production and removal (Brooks, 1986), i.e., the highest intensity point before the lactate concentration starts to increase during continuous exercise (Davis, 1985), called the maximal lactate steady state (MLSS). While the MLSS is an accurate method to determine LT, it is time consuming, and therefore a number of short-stage methods are more commonly used, such as a warm up value + 1.5 mmol⋅L^–1^ (Støren et al., 2014). LT may be expressed in absolute values, for example running velocity (LT_V_), or in relative values as a percentage of VO_2max_ (LT). In trained endurance athletes, LT usually represents an intensity between 75 and 90% of VO_2max_ (Joyner and Coyle, 2008).

In cycling, elite athletes have shown somewhat higher LT than regional athletes (Støren et al., 2014), which is in line with the expectations that well-trained endurance athletes in general have a higher LT than moderately trained athletes (Joyner and Coyle, 2008). However, McLaughlin et al. (2010) found nearly similar LT among runners at different performance levels.

In running, the most common way to express workload at LT seems to be as LT_V_ (Yoshida et al., 1987). LT_V_ has been found to primarily depend on VO_2max_ and work economy (C) in well-trained and elite endurance athletes (Yoshida et al., 1987; Bishop et al., 2000; Nicholson and Sleivert, 2001; Støren et al., 2008, 2013; McLaughlin et al., 2010). In previous studies, VO_2max_ divided by C has been defined as maximal aerobic speed (MAS) (Støren et al., 2008, 2012, 2014; Helgerud et al., 2010; Sunde et al., 2010, 2019; Bratland-Sanda et al., 2020; Johansen et al., 2020), and found to be a main determinant of aerobic endurance performance and LT_V_ (Støren et al., 2012, 2014; Sunde et al., 2019; Bratland-Sanda et al., 2020; Johansen et al., 2020). In accordance with the importance of MAS on LT_V_, elite long-distance runners have higher LT_V_ than national runners (Yoshida et al., 1987; Nicholson and Sleivert, 2001). Among runners with similar VO_2max_, a higher LT_V_ is typically due to a better C_R_ (Laye et al., 2015).

Several training interventions that have reported improved endurance running performance, also found improved LT_V_ (Ferrauti et al., 2010; Enoksen et al., 2011; Hottenrott et al., 2012). These improvements should therefore logically relate to either an improvement in VO_2max_, in C or in LT. However, studies reporting improved LT after training interventions among already well-trained endurance athletes are lacking. On the contrary, several studies have found no adaptations in LT after training interventions (Bangsbo, 1994; Helgerud et al., 2007; Støren et al., 2008; Sunde et al., 2010; Rønnestad et al., 2015). This implies that improvements in LT_V_ are most probably due to either an improvement in VO_2max_, an improvement in C, or both.

Studies assessing gender differences at the same relative performance level have found approximately 10–15% higher VO_2max_, and 10–15% higher LT_V_ in men than in women (Pollock, 1977; Pate et al., 1987; Helgerud, 1994; McLaughlin et al., 2010). Studies comparing C_R_ among men and women have found both better (Daniels and Daniels, 1992), similar (Bunc and Heller, 1989) and worse (Helgerud et al., 2010) C_R_ in men compared to women. In a study by Helgerud et al. (2010), female runners were found to have 10% better C_R_ than male runners, when C_R_ was expressed as ml⋅kg^–0.75^⋅m^–1^, which contradicts the findings of Daniels and Daniels (1992) and Bunc and Heller (1989). Few studies have reported gender differences in LT when expressed in relation to running performance, but Maldonado-Martin et al. (2004) found better LT in women marathon runners than in their male counterparts. It would thus be of interest to assess possible gender differences in LT or in the impact of LT_V_ determining variables.

In previous studies, the relative importance of LT_V_ on endurance performance have only been reported in relatively small cohorts. Studies on runners, such as Støren et al. (2008); Enoksen et al. (2011), Hottenrott et al. (2012) have not included more than 34 subjects at the most. In Støren et al. (2014), a large number of cyclists (*n* = 108) participated, enabling the calculation of a new model for predicting power output at LT (LT_W_) by using the following equation (Equation 1), where LT_W_ is the power output at LT, LT_%_VO_2max_ is LT_%_, and C_C_ is cycling economy (oxygen cost of cycling).

$$L\,T_{\text{w}}=\left(L\,T_{\%}\,V\,O_{2\,\max}\right)\,\frac{V\,O_{2\,\max}}{C_{\text{C}}}$$

The model correlated nearly perfect with measured LT_W_ (*r* = 0.98, *p* < 0.0001, SEE = 2.8%). This model for predicting LT_V_ is the same model as used for predicting performance in running distance races by McLaughlin et al. (2010). In short, the equation from Støren et al. (2014) is based on the product of LT and MAS. Given the proposition in Støren et al. (2014) that LT does not change in already well trained athletes, the first rationale to apply this equation also in runners is that it is a time-saving method to assess LT_V_. As proposed in Støren et al. (2014), a full LT profile test is then only needed once. For all subsequent tests, only MAS is needed. Further, if this equation applies also in running, this could be an argument to focus more on MAS, i.e., VO_2max_ and C_R_, and less on LT.

From both a practical and a theoretical point of view, it is of interest to identify relationships between LT_V_ and other physiological variables shown to affect endurance running performance. The aims of the present study were thus to investigate:

1. The impact of LT on LT_V_ in running, as well as the impact of VO_2max_ and C_R_ on LT_V_.
2. The fit of the equation for cyclists used in Støren et al. (2014) applied on runners.
3. Potential differences in LT and LT_V_ between elite, national and recreational runners.
4. Potential gender differences in point 1–3.

The hypothesis were that:

1. VO_2max_ and C_R_ would have a strong impact on LT_V_, while LT would not.
2. The equation for cyclists used in Støren et al. (2014) would fit also for runners.
3. Both LT and LT_V_ would be higher with higher performance level.
4. Male runners would have higher VO_2max_, C_R_, and LT_V_ than female runners, but similar LT.

## Materials and Methods

### Subjects

Seventy-five (37 male and 38 female) long-distance runners from Norway, with performance levels ranging from elite to regional, participated in the present study. Subject characteristics are presented in Tables 1, 2. Due to the large number of runners, we were not able to arrange a test-run over, e.g., 5000 m or 3000 m for all the runners. However, by collection of race results the same year as the physiological tests were performed, the range of performance was found to be from 8.05 to 13.30 min in 3000 m.

**TABLE 1:** Characteristics of runners by gender.

	**All (*n* = 75)**	**Men (*n* = 37)**	**Women (*n* = 38)**	***p* men vs. women**
BW (kg)	67.0 ± 11.0	76.1 ± 14.1	65.1 ± 5.6	<0.001
**VO_2max_**				
L⋅min^–1^	4.20 ± 1.00	5.08 ± 0.67	3.41 ± 0.47	<0.001
mL⋅kg^–1^⋅min^–1^	63.0 ± 9.3	67.1 ± 9.3	59.1 ± 7.5	<0.001
mL⋅kg^–0.75^⋅min^–1^	177.8 ± 30.0	191.6 ± 26.5	158.4 ± 18.3	<0.001
**C_R_**				
mL⋅kg^–1^⋅m^–1^	0.233 ± 0.019	0.235 ± 0.019	0.230 ± 0.019	0.336
mL⋅kg^–0.75^⋅m^–1^	0.664 ± 0.063	0.693 ± 0.058	0.636 ± 0.055	<0.001
**LT**				
%VO_2max_	83.6 ± 4.0	82.5 ± 4.0	84.6 ± 3.8	0.020
**LT_V_**				
km⋅h^–1^	13.6 ± 2.1	14.2 ± 1.9	13.1 ± 2.1	0.028
km⋅h^–1^ (pred.)	13.6 ± 1.7	14.0 ± 1.5	13.2 ± 1.7	0.028
MAS (km⋅h^–1^)	16.4 ± 2.7	17.2 ± 2.5	15.5 ± 2.6	0.006

*Values are mean ± SD. SD, standard deviation; BW, body weight; VO_2max_, maximal oxygen consumption; C_R_, oxygen cost of running; LT, lactate threshold; LT_V_, velocity at LT; MAS, maximal aerobic speed which is maximal oxygen consumption divided by oxygen cost of running.*

**TABLE 2:** Characteristics of runners by competitive level.

	**Elite (*n* = 12)**	**National (*n* = 29)**	**Recreational (*n* = 34)**	***p* Elite vs. National**	***p* Elite vs. Recreational**	***p* National vs. Recreational**
BW (kg)	61.9 ± 11.4	67.8 ± 11.2	68.2 ± 10.5	0.256	0.204	0.991
**VO_2max_**						
L⋅min^–1^	4.42 ± 1.15	4.62 ± 1.00	3.84 ± 0.85	0.828	0.178	0.006
mL⋅kg^–1^⋅min^–1^	71.2 ± 8.3	67.8 ± 6.2	56.1 ± 6.2	0.323	<0.001	<0.001
mL⋅kg^–0.75^⋅min^–1^	188.0 ± 39.0	193.2 ± 25.2	161.0 ± 20.8	0.827	0.008	<0.001
**C_R_**						
mL⋅kg^–1^⋅m^–1^	0.216 ± 0.022	0.232 ± 0.016	0.238 ± 0.018	0.018	<0.001	0.383
mL⋅kg^–0.75^⋅m^–1^	0.604 ± 0.066	0.665 ± 0.050	0.683 ± 0.060	0.007	<0.001	0.428
**LT**						
%VO_2max_	83.1 ± 4.1	82.4 ± 4.0	84.8 ± 3.9	0.859	0.431	0.053
**LT_V_**						
km⋅h^–1^	16.5 ± 1.6	14.4 ± 1.1	12.1 ± 1.0	<0.001	<0.001	<0.001
km⋅h^–1^ (pred.)	15.8 ± 0.7	14.4 ± 0.7	12.0 ± 1.2	<0.001	<0.001	<0.001
MAS (km⋅h^–1^)	19.8 ± 2.1	17.5 ± 1.4	14.1 ± 2.7	<0.001	<0.001	<0.001

*Values are mean ± SD. SD, standard deviation; BW, body weight; VO_2max_, maximal oxygen consumption; C_R_, oxygen cost of running; LT, lactate threshold; LT_V_, velocity at LT; MAS, maximal aerobic speed which is maximal oxygen consumption divided by oxygen cost of running.*

All subjects gave their written consent before participating, according to the regional ethical committee of the South-East of Norway. The study was approved by the institutional review board at USN.

### Design

The study was a cross-sectional multi-center study, aiming to identify factors influencing running velocity at lactate threshold in male and female runners at different levels of performance.

### Methodology

The subjects were tested in three different laboratories, at the University of South-Eastern Norway (USN), at Inland Norway University of Applied Sciences (INN), and at the Norwegian School of Sport Sciences (NSSS).

Test protocols were identical in all three laboratories. The same type of treadmill (Woodway PPS 55 Sport, Waukesha, Germany) and heart rate (HR) equipment (Polar Electro, Kempele, Finland) were used at all three locations. For lactate measurements, Arkray Lactate Pro LT-1710 analyzer (Arkray Inc., Kyoto, Japan) was used at USN and INN, while YSI 1500 Sports Lactate analyzer (Yellow Springs, Ohio, United States) was used at NSSS. Two different types of metabolic test systems were used; at USN, Sensor Medics V_max_ Spectra (Sensor Medics 229, Yorba Linda, CA, United States), and at INN and NSSS, Oxycon Pro (Erich Jaeger, Höchberg, Germany). The different systems were validated against each other for the range of measures that included all of the participants.

In the treadmill tests, the subjects started at a velocity of at least 3 km⋅h^–1^ below their expected LT_V_ (representing approximately 50% of VO_2max_). To account for this expectancy, the first velocity was adjusted if it did not represent 65–75% of maximal heart rate (HR_*max*_). All subjects knew their HR_*max*_ before the tests. Every 5 min, the running velocity increased by 1–1.5 km⋅h^–1^, until the test terminated just above the subjects’ LT.

The 5 min stages have in previous studies (Helgerud, 1994; Helgerud et al., 2010; Støren et al., 2008, 2013, 2014; Sunde et al., 2010) proved sufficient to reach steady state HR and VO_2_ in each step. To increase speed by 1–1.5 km⋅h^–1^ for each step, has in previous studies (Helgerud, 1994; Helgerud et al., 2010; Støren et al., 2008) proven adequate to reach LT in four to five steps.

Lactate threshold was defined as the warm up [La^–^]_b_ value (i.e., measured after the lowest running velocity) + 2.3 mmol⋅L^–1^, using the Arkray Lactate Pro LT-1710 analyzer (Arkray Inc., Kyoto, Japan). This is in accordance with the method of Helgerud (1994) and Helgerud et al. (2007), using the YSI 1500 Sports Lactate analyzer (Yellow Springs, Ohio, United States), with LT defined as the warm up [La^–^]_b_ value + 1.5 mmol⋅L^–1^. The difference in [La^–^]_b_ values between the two analyzers is constant, and due to the difference in [La^–^]_b_ values between hemolyzed blood (Arkray) and whole blood (YSI). Medbø et al. (2000) have reported the YSI to measure 67% of the lactate Pro values, meaning that 2.3 mmol⋅L^–1^ with lactate Pro equals 1.5 mmol⋅L^–1^ with YSI. Medbø et al. (2000) reported the exact same error of regression measured against enzyme photo fluorometry for the YSI and the lactate Pro. The LT assessment method in the present study was initiated by Helgerud et al. (1990), and has previously been used in several studies (Helgerud, 1994, Helgerud et al., 2010; Sunde et al., 2009; Støren et al., 2013, 2014). This method has also been recommended by Medbø et al. (2000). The main advantage of using individual warm up values plus a constant, compared to a fixed 4 mmol⋅L^–1^, is that it is less vulnerable to day to day variations in subjects [La^–^]_b_ (Støren et al., 2014). However, compared to a fixed model of 4 mmol⋅L^–1^, the model based on a warm-up [La^–^]_b_ value + 2.3 mmol⋅L^–1^, may result in lower LT_%_ and LT_V_ (Støren et al., 2014). In Støren et al. (2014) on cycling, the latter model resulted in a LT of 77%, while the use of a fixed 4 mmol⋅L^–1^ resulted in a LT of 81%.

A VO_2max_ test was performed 5–10 min after the LT assessment. An incremental protocol regarding velocity was used, with an elevation of the treadmill of 3%, 5% or 10%. The subjects started at a velocity representing LT_V_, rounded to the nearest km⋅h^–1^. Every 30 or 60 s, the velocity was increased by 0.5–1.0 km⋅h^–1^. This protocol was used to ensure a test duration between 3 and 8 min, as used in Støren et al. (2008). The test terminated at voluntary fatigue by the runners. A possible flattening of the VO_2_ curve (<1 ml⋅kg^–1^⋅min^–1^ increase in VO_2_ during the last three subsequent recordings), HR ≥ 95% HR_*max*_, respiratory exchange ratio (RER) ≥ 1.05, and [La^–^]_b_ ≥ 8.0 mmol⋅L^–1^ were used as criteria to evaluate if VO_2max_ was obtained.

Oxygen cost of running was calculated at the velocity representing 70% VO_2max_, as in Støren et al. (2008) and Helgerud et al. (2010). With several submaximal VO_2_ values at different submaximal running velocities in the LT assessment test, and with the VO_2max_ value from the VO_2max_ test, this was possible. The submaximal running velocities were plotted against the corresponding VO_2_ values, representing a linear regression with *r*–values never below 0.99. From this linear regression, 70% of VO_2max_ was plotted, representing a running velocity. By dividing 70% of VO_2max_ by this velocity, e.g., 40 mL⋅kg^–1^⋅min^–1^ /200 m⋅min^–1^, C_R_ was expressed as the oxygen cost of running per meter, e.g., 0.200 ml⋅kg^–1^⋅m^–1^.

Maximal aerobic speed was set as the velocity point where the horizontal line representing VO_2max_ meets the extrapolated linear regression representing the submaximal VO_2_ measured in the LT assessment. The linearity from this regression in previous studies (Støren et al., 2008, 2014; Helgerud et al., 2010; Sunde et al., 2010) has been reported to be very good (*r* > 0.99, *p* < 0.001). This method to define MAS implies that MAS equals VO_2max_ /C_R_. Since VO_2max_ may be expressed as mL⋅kg^–1^⋅min^–1^, and C_R_ as mL⋅kg^–1^⋅min^–1^, VO_2max_ /C_R_ is expressed as m⋅min^–1^, i.e., a velocity.

Maximal oxygen uptake values were expressed in both mL⋅kg^–1^⋅min^–1^, mL⋅kg^–0.75^⋅min^–1^, and in L⋅min^–1^ as proposed by Bergh et al. (1991). The importance of allometric scaling in running was shown by Helgerud (1994).

A mathematical model for predicting LT_V_ (Equation 2), based on the equation in Støren et al. (2014) was used in the present study:

$$L\,T_{\text{V}}=\left(L\,T_{\%}\right)\,\frac{V\,O_{2\,\max}}{C_{\text{R}}}$$

LT_V_ is the running velocity at LT, LT_%_ is LT in percent of VO_2max_, and C_R_ is the oxygen cost of running.

### Statistical Analysis

Data were tested for normality by use of QQ-plots and the Kolmogorov Smirnov test, and found to represent a normal distribution for VO_2max_, C_R_ and LT. All table values were therefore expressed descriptively as mean ± standard deviation (SD), or the correlation factor *r* with confidence intervals. The Pearson Bivariate two-tailed correlation test was used to determine correlations between variables. The correlation coefficient definitions by Hopkins et al. (2009) where *r* values of 0.3–0.5 = moderate, 0.5–0.7 = large, 0.7–0.9 = very large, 0.9 = nearly perfect, and 1.0 = perfect, were used to describe correlations. A General Linear Model (GLM) with Tukey *post hoc* test was performed to detect possible differences between the three groups divided by competition level. Statistical analyses were performed using the software program SPSS, version 19.0 (Statistical Package for Social Science, Chicago, IL, United States). A *p*-value < 0.05 was accepted as statistically significant for all tests, including between group differences and correlations.

## Results

Characteristics of runners by competitive level are shown in Table 2. Scaled for body mass to the power of 0.75, the elite runners had 17% higher VO_2max_ than the recreational runners (*p* = 0.01), but there was no difference between the elite and the national runners. However, a GLM showed increasing VO_2max_ with higher performance level overall (*p* < 0.001). The elite runners had 10% better C_R_ than the national runners (*p* < 0.001). There was no difference in C_R_ between national and recreational runners, but the GLM showed better C_R_ with higher performance level overall (*p* < 0.001). There were no differences between the three performance levels regarding LT, but the elite runners had 15% better LT_V_ than the national runners (*p* < 0.001), who in turn had 20% better LT_V_ than the recreational runners (*p* < 0.001). Also, GLM showed increasing LT_V_ with higher performance level overall (*p* < 0.001). MAS increased with increasing performance level (*p* < 0.001), where elite runners had 13% higher MAS than national runners (*p* < 0.001) who in turn had 24% higher MAS than recreational runners (*p* < 0.001).

Characteristics of runners by gender are shown in Table 1. Expressed as mL⋅kg^–0.75^⋅min^–1^, the male runners had 21% higher VO_2max_ (*p* < 0.001), but 9% poorer C_R_ than the female runners (*p* < 0.001). The female runners had 2.5 percentage point higher LT (*p* = 0.020), but 8% lower LT_V_ than the male runners (*p* = 0.028).

Regardless of competitive level or gender, the mathematical model (Equation 2) showed that LT_V_ was almost identical to the measured LT_V_ (13.6 ± 2.1 vs. 13.6 ± 1.7 km⋅h^–1^, *p* = 0.78).

**FIGURE 1:**
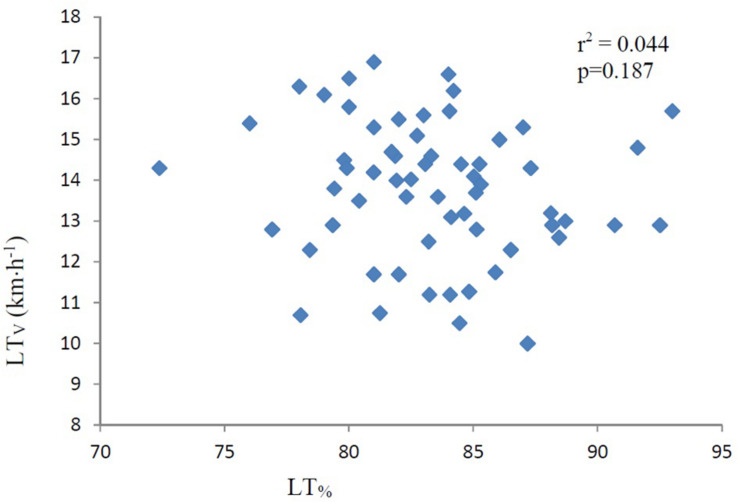
LT vs. LT_V_ (km⋅h^–1^). The figure also displays the determining *r* (*r*^2^). LT, lactate threshold in percent of maximal oxygen consumption (VO_2max_). LT_V_, running velocity at lactate threshold. km⋅h^–1^, kilometers per hour. The correlation is not significant (*p* = 0.187).

**FIGURE 2:**
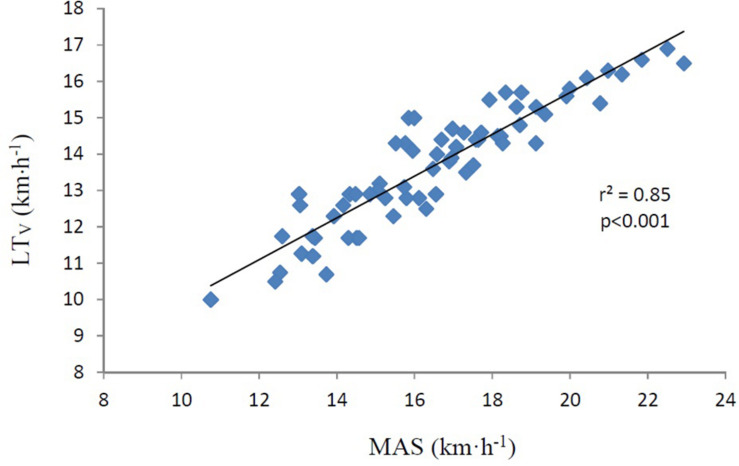
MAS (km⋅h^–1^) vs. LT_V_ (km⋅h^–1^). The figure also displays the determining *r* (*r*^2^). MAS, maximal aerobic speed, which is maximal oxygen consumption divided by oxygen cost of running. LT_V_, running velocity at lactate threshold. km⋅h^–1^, kilometers per hour. The correlation is significant (*p* < 0.001).

**FIGURE 3:**
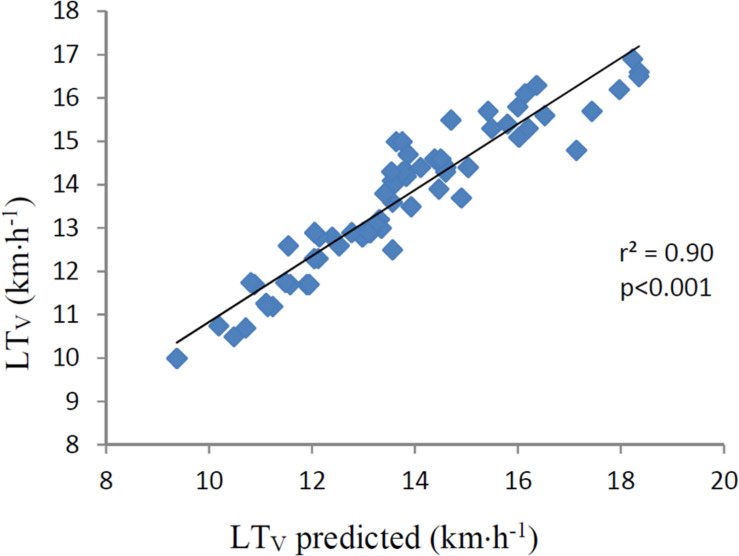
LT_V_ (km⋅h^–1^) predicted vs. LT_V_ (km⋅h^–1^). The figure also displays the determining r (r^2^). Predicted LT_V_ is the product of individual LT_%_ and MAS. LT, lactate threshold in percent of maximal oxygen consumption (VO_2max_). LT_V_, running velocity at lactate threshold. km⋅h^–1^, kilometers per hour. MAS, maximal aerobic speed, which is maximal oxygen consumption divided by oxygen cost of running. The correlation is significant (*p* < 0.001).

Correlations are presented in Table 3. LT did not correlate with LT_V_ (*r* = −0.15, *p* = 0.187) as presented in Figure 1. MAS, calculated by VO_2max_/C_R_, correlated nearly perfect (*r* = 0.92 *p* < 0.001) with LT_V_ (Figure 2). The product of LT and MAS (Equation 2), correlated nearly perfect (*r* = 0.95 *p* < 0.001) with LT_V_ (Figure 3). Based on the *r*^2^ of 0.90 between calculated and measured LT_V_, and the *r*^2^ of 0.85 between MAS and measured LT_V_, the relative importance of LT on LT_V_ was 0.90 – 0.85 = 5%.

**TABLE 3:** Relationship between different variables and running velocity at the lactate threshold.

	**All, *n* = 75**	***p***	**Males, *n* = 37**	***p***	**Females, *n* = 38**	***p***
LT	−0.15 (−0.34,0.04)	0.187	−0.15 (−0.42,0.12)	0.366	−0.04 (−0.31,0.23)	0.822
**Anthropometrics**						
BW (kg)	0.03 (−0.16,0.22)	0.831	−0.30 (−0.57, −0.03)	0.070	−0.22 (−0.49,0.05)	0.038
**VO_2max_**						
L⋅min^–1^	0.51 (0.32,0.70)	<0.001	0.67 (0.40,0.94)	<0.001	0.41 (0.14,0.68)	0.010
mL⋅kg^–1^⋅min^–1^	0.79 (0.60,0.98)	<0.001	0.81 (0.54,1.08)	<0.001	0.78 (0.51,1.05)	<0.001
mL⋅kg^–0.75^⋅min^–1^	0.64 (0.45,0.83)	<0.001	0.80 (0.53,1.07)	<0.001	0.50 (0.23,0.77)	<0.001
**C_R_**						
mL⋅kg^–0.75^⋅m^–1^	−0.34 (−0.53, −0.15)	0.003	−0.37 (−0.64, −0.10)	0.023	−0.67 (−0.94, −0.40)	<0.001
**MAS**						
VO_2max_/C_R_	0.92 (0.73,1.11)	<0.001	0.93 (0.66,1.20)	<0.001	0.91 (0.64,1.18)	<0.001
LT⋅ (VO_2max_/C_R_)	0.95 (0.76,1.14)	<0.001	0.95 (0.68,1.22)	<0.001	0.94 (0.67,1.21)	<0.001

*Values are *r*, and confidence interval in parenthesis. BW, Body weight; Kg, kilogram; VO_2max_, maximal oxygen uptake; L, liters; Kg, kilograms; mL, milliliters; min, minutes; m, meters; CR, oxygen cost of running; MAS, maximal aerobic speed which is maximal oxygen consumption divided by oxygen cost of running; LT, LT in percent of maximal oxygen consumption; LT_V_, running velocity at LT.*

VO_2max_ (*r* = 0.64, *p* < 0.001) and C_R_ (*r* = 0.34, *p* = 0.003) showed large and moderate correlations, respectively, with LT_V_.

There were no gender differences in correlations between LT_V_ and the other physiological variables, with one exception. The female runners had a stronger correlation between LT_V_ and C_R_ (*r* = 0.67, *p* < 0.001) than the male runners (*r* = 0.37, *p* = 0.023).

## Discussion

The main findings in the present study were that:

1. LT did not correlate with LT_V_, neither when the runners were divided by competitive level, by gender, or when all runners were taken together in one group. Both VO_2max_ and C_R_ correlated separately with LT_V_, but the strongest correlation with LT_V_ was when VO_2max_ was divided by C_R_, expressing MAS.
2. The product of LT and MAS (Equation 2), expressed an even stronger correlation with LT_V_, indicating a good fit of the equation presented in Støren et al. (2014).
3. There were no differences between elite, national and recreational runners regarding LT, but LT_V_, VO_2max_ and C_R_ were found to be higher with higher performance level.
4. Female runners had slightly higher LT than the male runners. Male runners had higher VO_2max_, but not as good C_R_ as the female runners. The female runners had a stronger correlation between LT_V_ and C_R_ than the male runners.

### LT as a Percent of VO_2max_

Lactate threshold was not different between elite, national and recreational runners in the present study. Elite runners would most probably have performed a large amount of endurance training to reach their level. If extensive aerobic endurance training were to improve LT substantially, we would expect elite runners to have a higher LT than less competitive runners. We have found no intervention studies reporting improved LT after endurance training in already well-trained endurance athletes, but several reports of no adaptations in LT (Bangsbo, 1994; Helgerud et al., 2007; Støren et al., 2008; Sunde et al., 2010; Rønnestad et al., 2015).

The finding that LT in the present study did not correlate with LT_V_, supports the assumption that LT is not a major determinant of aerobic endurance performance. This is in accordance with McLaughlin et al. (2010) who did not find a correlation between LT and endurance race time performance. The present results are not entirely in line with previous results from cycling (Støren et al., 2014), where a correlation between LT and LT_V_ was found. However, LT was in Støren et al. (2014) found to be a poor determinant, -and explained only 15% of the variability in work load at LT.

The 2.5 percentage point better LT in the female runners as opposed to the males in the present study could of course be by coincidence, as it is a minor difference in this context. On the other hand, with as many as 38 females and 37 males, the statistical power is quite strong. Few studies have reported gender differences in LT, but Maldonado-Martin et al. (2004) found women marathon runners to have better LT than their male counterparts, thus in accordance with the present results. It may be argued that two short-stage methods to determine LT, as used in the present study, is not as accurate as the determination of MLSS based on several stages of 20–30 min of continuous work (Faude et al., 2009). However, the two different methods used in the present study are both based on the work by Helgerud et al. (1990) resulting in a warm up value + 1.5 mmol⋅L^–1^, and found to represent MLSS within the warm up value + 1.3 – 1.7 mmol⋅L^–1^.

### LT Velocity

Running velocity at LT was found to be higher at higher performance levels in the present study. This is in line with several studies on both running and cycling where LT_V_ has been associated with aerobic endurance performance (Yoshida et al., 1987; Helgerud, 1994; Bishop et al., 2000; Nicholson and Sleivert, 2001; Støren et al., 2008, 2013, 2014; McLaughlin et al., 2010). Based on the nearly perfect fit between Equation 2 and LT_V_, it was natural to assume that the main determinants for LT_V_ was LT, VO_2max_, and C_R_. Further, as LT did not correlate with LT_V_ in the present study, it had probably very little impact on the differences in LT_V_ between the different performance levels. This is further supported by the lack of difference in LT between these three groups. VO_2max_, on the other hand was found to be higher in elite than in recreational runners, and C_R_ was found to be better in elite than in national and recreational runners in the present study. The finding that both VO_2max_ and C_R_ correlated with LT_V_ was therefore not surprising. In both male and females, VO_2max_ determined LT_V_ with approximately 45–50% based on *r*^2^ – values, which was about three times the determining factor for C_R_. Although VO_2max_ and C_R_ correlated separately with LT_V_, the strongest correlation with LT_V_ was when VO_2max_ was divided by C_R_, expressing MAS. Based on the *r*^2^ –value, MAS predicted LT_V_ by 85%. This is in accordance with McLaughlin et al. (2010), finding that the product of VO_2max_ and C_R_ correlated by *r* = 0.97 (*r*^2^ = 0.94) with endurance race time performance. In the present study, we used the same equation for predicting LT_V_ as in cycling in Støren et al. (2014) (Equation 2). Interestingly, results from the present study expressed the same nearly perfect correlation between predicted and measured LT_V_ as in Støren et al. (2014). With an *r* –value of 0.95 and a SEE of 4.0%, the product of MAS and individual LT thus explained 90% of LT_V_, outside a range of ±0.27 km⋅h^–1^. These results may indicate that the equation for calculating LT_W_ in cycling (Støren et al., 2014) can be used also in running (LT_V_). This model is time saving. For all LT_V_ tests following the first initial LT assessment, the time spent per test would be less than half of a full incremental LT_V_ test. This model only need capillary blood samples during the first initial LT assessment. From the second test on, no blood samples are needed.

### VO_2max_ and Cost of Running

The runners in the present study were representing a heterogeneous group regarding VO_2max_ and C_R_, with a CV of 17% and 9%, respectively. The material in the present study was thus slightly more heterogeneous than the material in McLaughlin et al. (2010). Based on *r*^2^ –values in the present study, VO_2max_ and C_R_ predicted 62% and 12% of LT_V_, respectively. In a more homogeneous group regarding VO_2max_, C_R_ would probably be of greater importance, as shown in Conley and Krahenbuhl (1980). However, the female runners in the present study had both a 9% better C_R_, and a better correlation between LT_V_ and C_R_ than the male runners. This is not in accordance with McLaughlin et al. (2010) reporting better C_R_ among the male runners. Also, Daniels and Daniels (1992) reported male runners to have approximately 7% better C_R_ than female runners. However, neither McLaughlin et al. (2010) or Daniels and Daniels (1992) scaled for body weight raced to the power of 0.75, as in the present study. If not allometrically scaled, C_R_ in the present study was not significantly different between males and females. This underlines the importance of allometric scaling when comparing male and female runners. The determining factor (based on the *r*^2^ – values) of C_R_ on LT_V_ in the present study, was more than three times higher for the female than the male runners. The latter cannot be explained by a smaller CV in VO_2max_ in the female runners. The better C_R_ in the female runners was in accordance with the results from Helgerud et al. (2010). As only six female runners participated in the study by Helgerud et al. (2010), the generalizing value is apparently low, although strengthened by the similar results from the present study. Why female runners may have a better C_R_ than male runners of the same relative competitive level is a question with no apparent answer.

## Practical Implications

From the results of the present study, we suggest that the same equation previously used in cycling also could be used in running. The present results further imply that training to improve LTv should be focused toward increasing VO_2max_ and or C_R_. In order to accomplish such improvements, we propose high intensity aerobic interval training (Østerås et al., 2002; Helgerud et al., 2007; Støren et al., 2012) in order to improve VO_2max_, and maximal strength training (Østerås et al., 2002; Støren et al., 2008; Sunde et al., 2010) to improve C_R_. As LT_V_ seemed to be dependent on MAS and not LT_%_, we propose the testing of MAS rather than LT in order to evaluate training achievements in competitive runners.

## Conclusion

Lactate threshold was not related to LT_V_ or performance level in the present study. As hypothesized, VO_2max_ and C_R_ were found to be better with increasing performance level, and correlated well with LT_V_. This highlights the importance of VO_2max_ and C_R_ on LT_V_. LT was not found to be higher with higher performance levels, as opposed to the hypothesis. There were no gender differences in the relative impact of the factors determining LTv, with one exception. C_R_ seemed to have a larger impact on LT_V_ in females compared to males. Also, the females had a higher LT and a better C_R_ than their male counterparts. The equation for assessing LT_V_ previously used in cycling was found applicable also for assessing LT_V_ in running as hypothesized. We propose testing of MAS, rather than LT when evaluating training achievements in competitive runners of both genders.

## Data Availability Statement

The raw data supporting the conclusions of this article will be made available by the authors, without undue reservation.

## Ethics Statement

The studies involving human participants were reviewed and approved by the Institutional Review Board at USN. The patients/participants provided their written informed consent to participate in this study.

## Author Contributions

ES, ØS, and JaH designed and planned the project. The interpretation of the data was led by ØS. ES led the writing of the manuscript. All authors took part in the data collection, as well as contributing in the different parts of data analyses, and edited, reviewed and approved the final manuscript.

## Conflict of Interest

The authors declare that the research was conducted in the absence of any commercial or financial relationships that could be construed as a potential conflict of interest.
